# Application of a Heuristic Framework for Multilevel Interventions to Eliminate the Impact of Unjust Social Processes and Other Harmful Social Determinants of Health

**DOI:** 10.1007/s11121-024-01658-x

**Published:** 2024-04-12

**Authors:** Vincent Guilamo-Ramos, Marco Thimm-Kaiser, Adam Benzekri, Celia Johnson, Desiree Williams, Nash Wilhelm-Hilkey, Melody Goodman, Holly Hagan

**Affiliations:** 1 Center for Latino Adolescent and Family Health, Johns Hopkins University, Washington, DC 20001 USA; 2 Institute for Policy Solutions, Johns Hopkins University School of Nursing, Johns Hopkins University, Washington, DC 20001 USA; 3 School of Nursing, Johns Hopkins University, Baltimore, MD 21205 USA; 4 Presidential Advisory Council On HIV/AIDS, US Department of Health and Human Services, Washington, DC USA; 5 Department of Epidemiology, School of Global Public Health, New York University, New York, NY 10003 USA; 6 Department of Biostatistics, School of Global Public Health, New York University, New York, NY 10003 USA; 7 Department of Social Behavioral Sciences, School of Global Public Health, New York University, New York, NY 10003 USA

**Keywords:** Conceptual framework, Mechanism-focused multilevel intervention research, Social determinants of health, Structural racism, Meaningful community engagement

## Abstract

There is consensus about the importance of developing a strong cadre of effective multilevel interventions to eliminate the impacts of unjust social processes, such as structural racism and other harmful social determinants of health (SDOH), on health inequities in the USA. However, the available cadre of rigorously evaluated evidence-based interventions for SDOH mitigation remains underdeveloped relative to the magnitude of historic and current health inequities. The proposed manuscript addresses this gap in two ways: first, by introducing a heuristic framework to inform decisions in multilevel intervention development, study design, and selection of analytic methods and, second, by providing a roadmap for future applications of the framework in multilevel intervention research through an exemplar application using the ongoing NIH-funded evaluation study of the Nurse-Community-Family Partnership (NCFP) intervention. NCFP leverages individual, family, institutional, and system factors to shape COVID-19 mitigation outcomes at the individual and household levels. NCFP takes an approach informed by the heuristic framework to addressing and mitigating unjust social processes and other harmful SDOH. We discuss the application of a two-arm parallel explanatory group randomized trial to evaluate the efficacy of NCFP in improving the primary (COVID-19 testing uptake) and secondary (adoption of COVID-19 control measures, COVID-19 vaccine uptake, mutual aid capacity, etc.) outcomes at the individual and household levels. The analysis approach relies on random-intercept models, and we calculate the variance partitioning coefficient to estimate the extent to which household- and individual-level variables contribute to the outcome, allowing examination of NCFP effects at multiple levels.

**Supplementary Information:**

The online version contains supplementary material available at 10.1007/s11121-024-01658-x.

## Introduction

Despite having significantly higher per capita healthcare expenditures than other high-income countries, the USA consistently achieves among the worst population health outcomes (Gunja et al., 2022). Health inequities, which are larger in the USA than in peer countries (Schneider et al., 2021), contribute substantially to these unsustainably high healthcare costs and poor health outcomes (Davis et al., 2022; Dwyer-Lindgren et al., 2017; LaVeist et al., 2011). If unaddressed, the annual costs of health inequities in the USA are projected to triple by 2040, increasing to upwards of $1 trillion (Davis et al., 2022).

Health inequities are in large part shaped by harmful social determinants of health (SDOH)—systematic, unjust, and avoidable differences in the conditions in which people are born, grow, work, live, and age (Weinstein et al., 2017)—with structural racism representing a particularly well-documented adverse social process shaping inequitable health outcomes in communities of color (Benjamin et al., 2022; Gaskin et al., 2004; LaVeist, 2000; Shim & Compton, 2018). The role of unjust social processes, such as structural racism, xenophobia, and homophobia, and other SDOH as key drivers of health inequities has been recognized for decades. Despite this longstanding recognition, there has been limited progress in reducing health inequities in the USA. For example, county-level disparities in life expectancy—approximately three-quarters of which were explained by factors associated with harmful SDOH—have steadily increased between 1980 and 2014 (Dwyer-Lindgren et al., 2017). Relative to the magnitude of these historic and current health inequities, the cadre of rigorously evaluated and evidence-based interventions for SDOH mitigation remains underdeveloped (Williams et al., 2021).

The notable lack of progress in reducing and mitigating the health impacts of harmful SDOH over the past decades indicates that new and more effective tools for advancing health equity are sorely needed. Multilevel interventions have been recognized as particularly suitable and promising opportunities for addressing the complex, non-linear, multilevel, and dynamic SDOH mechanisms that shape health inequities (Alegria et al., 2021; Agurs-Collins et al., 2019; Paskett et al., 2016; Smedley, 2019). However, no consensus on the optimal conceptual and methodological approaches to multilevel intervention research has been reached (Alegria et al., 2021; Agurs-Collins et al., 2019; Paskett et al., 2016). Therefore, synthesizing overarching frameworks that guide intervention development and evaluation is an important area of scholarship. We will advance this work in two ways: first, by introducing a heuristic framework to inform decisions in multilevel intervention development, study design, and selection of analytic methods and, second, by providing a roadmap for future applications of the framework in multilevel intervention research through an exemplar application using the ongoing NIH-funded evaluation study of the Nurse-Community-Family Partnership (NCFP) intervention.

## A Heuristic Framework for Multilevel SDOH Intervention Research

A large body of conceptual and empirical work has advanced the scientific understanding of SDOH as the underlying drivers of health inequities. Several influential SDOH theories have emerged (Jones et al., 2019; Krieger, 2012; Link & Phelan, 1995; Rhodes, 2009; Singer & Clair, 2003; World Health Organization [WHO], 2010), and various frameworks capture aspects of SDOH influence, such as the widely cited SDOH framework developed by the WHO (2010). However, a synthesis of this work into a parsimonious, generalizable, and practice-oriented conceptual roadmap for the development and evaluation of multilevel SDOH interventions remains needed to promote translation of this literature into effective public health practice.

**Fig. 1:**
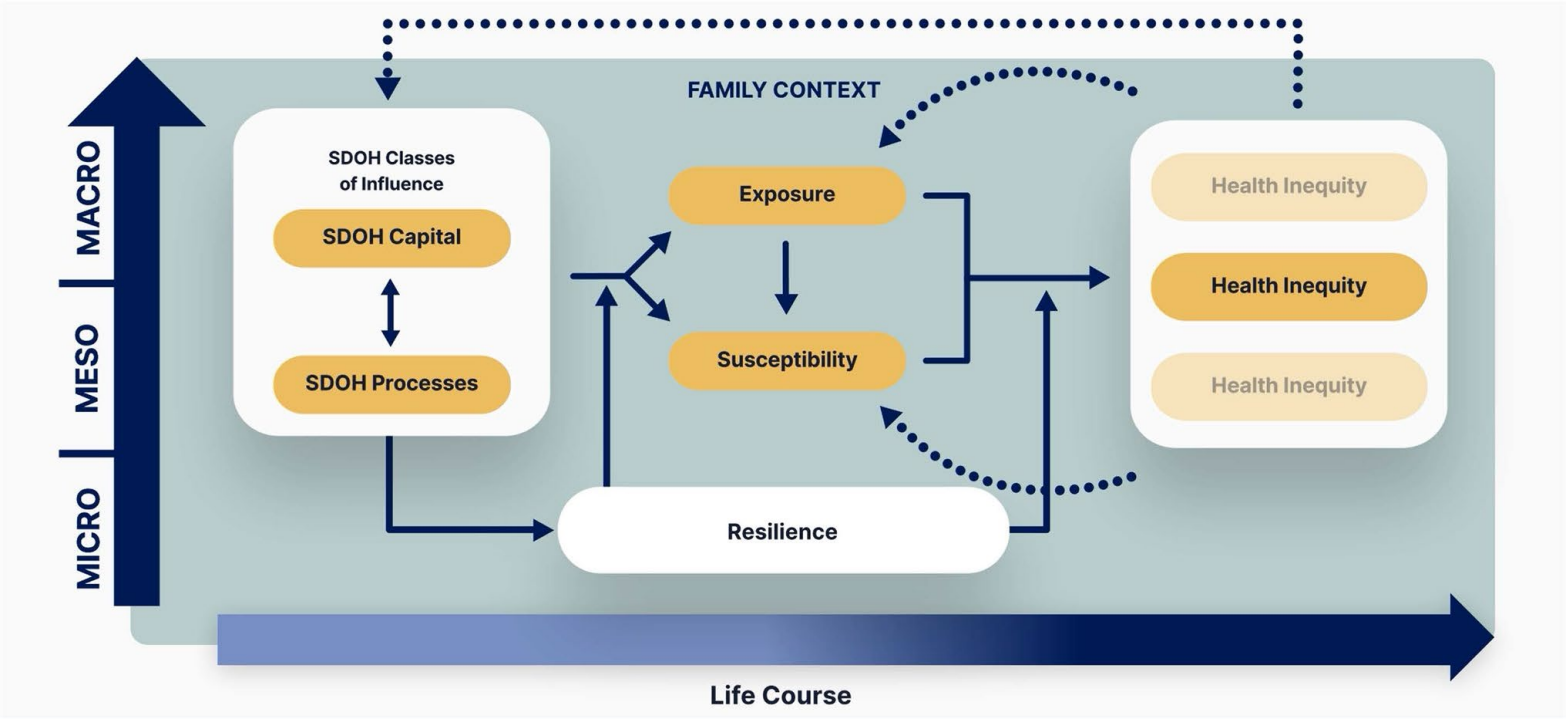
The CLAFH framework for mitigating harmful SDOH

To this end, this manuscript introduces and applies the Center for Latino Adolescent and Family Health (CLAFH) framework of SDOH mechanisms. The framework, depicted in Fig. 1, represents a comprehensive yet generalizable template of core SDOH constructs and mechanisms that warrant consideration during the development and evaluation of theory-based multilevel SDOH mitigation programs. The framework is designed for use with a range of different health inequities, and it therefore accommodates broad classes of SDOH-related constructs and dynamic relationships, which need to be operationalized more narrowly during application to a specific health inequity and context (see the “Four Steps for Applying the Framework” section below). Overall, the framework provides researchers with a template for critically appraising, conceptualizing, and operationalizing a series of core SDOH processes and mechanisms in relation to a specific context and health inequity for the purpose of creating a mental map of context-specific inequity drivers, which can subsequently inform decisions in study design, intervention development and evaluation, selection of analytic methods, etc. We briefly introduce the framework’s main components in the following paragraphs (bolded). In addition, Table 1 defines each key construct and provides an example operationalization. The framework’s development draws on a synthesis of key principles from a review of landmark SDOH theories and research and is discussed in depth elsewhere, along with detailed guidance for application (Guilamo-Ramos et al., 2023; Thimm-Kaiser et al., 2023).

**Table 1:** Definitions and exemplar operationalizations for key framework constructs

**Definition of key framework constructs**	**Example operationalization for COVID-19 inequities in the South Bronx, NYC**
**Health inequity:** a health disparity that is assumed to be produced through systematic, unfair, and avoidable causal mechanisms; health inequities tend to cluster/co-occur in specific spatiotemporal contexts	*Primary:* elevated community COVID-19 morbidity and mortality *Syndemic:* elevated community prevalence of cardiovascular disease
**SDOH capital:** resources and opportunities that are socially allotted to persons or groups and that affect health outcomes (e.g., income, educational attainment, quality of housing, accessibility, and quality of available healthcare)	*Macro:* mostly low-square footage, poorly ventilated housing available *Meso:* lack of feasible public health guidance for households in resource-limited settings *Micro:* financial dependence on continued in person, face-to-face “essential” work
**SDOH processes:** social factors shaping the interactions between persons, groups, institutions, or systems that affect health outcomes (e.g., racism, sexism, classism, xenophobia, and homophobia)	*Macro:* low societal valuation of Latino and Black “essential” workers’ health and wellbeing *Meso:* cultural/linguistic misalignment of social and economic support services *Micro:* sociocultural influences (religiosity, acculturation, *machismo*, *familismo*, etc.)
**Exposure:** subjection to a health risk or protective factor (exposure is environmental or behavioral)	High density of housing; inadequacy of social distancing/isolation opportunities
**Susceptibility:** the likelihood of morbidity/mortality, given the exposure to a health risk factor (susceptibility is biological)	Physiological stress-response system (neuro, endocrine, etc.); preexisting health conditions increase the risk of severe COVID-19
**Resilience**: collective action to reduce the harmful impact of structural adversity on individuals, institutions, and communities/populations’ ability to thrive (resilience does not solely rely on the strengths of individuals and communities and requires societal investments in the multilevel systems responsible for advancing health equity)	*Micro:* family capacity for COVID-19 mitigation *Meso:* responsiveness of health and public health infrastructure within the community *Macro:* political responsiveness and alignment of resources with community need

***Health inequities*** tend to cluster in communities affected by harmful SDOH. Because co-occurring health inequities interact, one health inequity can seldom be addressed in isolation. Drawing on syndemic theory (Singer & Clair, 2003), the framework seeks to guide multilevel intervention researchers toward considering and addressing the health of a community or population comprehensively and holistically. To this end, the framework depicts a spatiotemporal context of several co-occurring and clustered health inequities as the outcome of interest.

The CLAFH framework conceptualizes exposure and susceptibility as key mediators through which the impacts of SDOH operate and argues that they warrant consideration by multilevel intervention researchers as possible leverage points for SDOH mitigation. ***Exposure*** refers to the presence (or absence) of environmental and behavioral health risks and protective factors.

***Susceptibility*** represents the process by which social and structural conditions initiate and sustain biological changes (e.g., epigenetic, neurodevelopmental, immune, endocrine, and microbiome) that affect the likelihood of negative physical or mental health outcomes (Hertzman & Wiens, 1996). While these exposures and susceptibilities in a given community constitute the immediate drivers of health inequities, their population-level distributions are shaped by structural forces (Krieger, 2012; Phelan et al., 2010). By comprehensively targeting multiple inequity drivers, multilevel interventions are uniquely positioned to simultaneously address proximal exposure and susceptibility factors, alongside underlying SDOH influences (Agurs-Collins et al., 2019).

The framework distinguishes between two reciprocally related classes of SDOH influence as underlying drivers of health inequities: (1) ***SDOH capital***, defined as resources and opportunities that are socially allotted to persons or groups and that affect health outcomes (e.g., income, quality of housing and accessibility and quality of available healthcare), and (2) ***SDOH processes***, defined as social factors that shape the interactions between persons, groups, institutions, or systems that affect health outcomes (e.g., racism, sexism, classism, xenophobia, and homophobia).

SDOH capital and processes—many of which are unjust—shape health inequities, both together and independently. For example, the frequency and quality of interactions with the healthcare system are shaped by access to resources and opportunities, such as health insurance coverage or transportation options to the nearest primary care provider, and social processes, such as discrimination or stigmatization within healthcare settings. Therefore, if conceptualized separately, they offer different opportunities for mitigation.

The framework also accommodates multiple levels of influence. SDOH mechanisms operate non-linearly through the interplay of ***macro, meso, and micro influences***, all of which interact to reinforce or weaken each other’s effects (Berkman & Lochner, 2002). Macro variables include structural influences such as economic conditions and societal sentiment; meso variables include institutional influences such as healthcare settings and schools; and micro variables concern individual characteristics such as cultural background and behaviors. All constructs/relationships in the CLAFH framework can be conceptualized as macro, meso, or micro influences.

The impacts of SDOH have also been shown to accumulate over the ***life course*** (Jones et al., 2019). Moreover, health inequities attributable to harmful SDOH tend to cluster across generations (Weinstein et al., 2017). At the same time, the potential of families to act as powerful protective buffers against environmental risk is well established (Jones et al., 2019). Thus, the framework encourages a developmental perspective on SDOH impact and emphasizes the ***family as a primary context*** in which SDOH mechanisms operate. Focusing on the family unit as a primary context for shaping individual health outcomes is an underrecognized opportunity for SDOH interventions, particularly for multilevel interventions designed to go beyond consideration of the individual for intervention delivery and outcome ascertainment.

Finally, the CLAFH framework emphasizes the value of promoting multilevel ***resilience***. The strength-based factors that reduce the harmful impact of structural adversity on individuals, institutions, and communities/populations’ ability to thrive remain too often overlooked (noting that resilience does not solely rely on the strengths of individuals and communities and requires collective action and societal investments in the multilevel systems that are responsible for advancing health equity). Identifying and understanding the macro-, meso-, and micro-level resilience factors is a major opportunity to enhance efforts to reduce health inequities.

### Four Steps for Applying the Framework

Application of the framework to inform decisions in multilevel intervention development, study design, and selection of analytic methods involves four steps. In step 1, multilevel intervention researchers and community stakeholders jointly select the primary health inequity and geographic context to which the framework will be applied. Notably, meaningful community engagement (MCE) and collaboration throughout research development, implementation, analysis, and dissemination strengthen the delivery and impact of multilevel interventions. Evidence-based frameworks and principles for MCE involving researchers, community partners, and other stakeholders exist, such as the conceptual model developed by the Organizing Committee for Assessing Meaningful Community Engagement in Health & Health Care Programs & Policies (2022) or the consensus principles developed by Goodman et al. (2020). In addition, formal assessments of MCE, e.g., using the Research Engagement Survey Tool (Goodman et al., 2016), provide valuable yet too seldomly collected insights during multilevel intervention development and evaluation (Hudson et al., 2023; McCloskey et al., 2011).

In step 2, researchers operationalize each of the framework’s constructs and relationships relative to the specified health inequity of interest using theory, empirical data, and the published literature. Specifically, researchers identify co-occurring syndemic health inequities within the selected community context; exposure and susceptibility factors that directly contribute to the selected priority health inequity; underlying SDOH capital and process factors that elevate exposure to health risk factors, reduce exposure to protective factors, and increase susceptibility; and strength-based resilience factors that can offset the impacts of harmful SDOH. Macro-, meso-, and micro-level influences warrant consideration during the operationalization of all framework constructs and relationships, as well as developmental life course considerations and family influence. MCE that incorporates the lived experiences of community members during framework operationalization is key to ensure that identified SDOH mechanisms and intervention leverage points are consistent with practice-based evidence and have real-world utility.

In step 3, researchers and their community collaborators draw on the operationalized framework to identify leverage points that address and mitigate harmful SDOH impacts and to specify the corresponding components of the multilevel intervention package. Researchers review each factor in their hypothesized causal network of SDOH mechanisms (the operationalized framework) to evaluate whether it can reasonably be altered within the scope and available resources of the respective research project. Factors deemed amenable to change represent worthwhile leverage points for intervention. The identified leverage points, in turn, provide insights about the substantive and methodological expertise needed to develop and evaluate the multilevel intervention package. MCE enhances intervention development by incorporating community-derived theories of change and solutions into the intervention package.

In step 4, researchers evaluate the multilevel intervention package, drawing on their formative research in steps 1–3, with the operationalized framework and corresponding intervention package representing a roadmap to inform study design decisions. The leverage points to be targeted by the intervention package represent potential primary and secondary evaluation outcomes. In addition, the operationalized framework can inform other aspects of study design (e.g., defining the unit of intervention delivery and randomization (group vs. individual)), study measurement and analysis (e.g., selection of covariates for model specification and theory-based explanatory mediation analyses of intervention effects), and power considerations (e.g., number of groups per condition and group size). The selection and specification of appropriate analytic approaches require statistical guidance, and researchers may consider consulting available frameworks for the identification of statistical parameters, such as Petersen’s causal roadmap (2014) or the estimand framework for clinical trials (ICH E9[R1] Expert Working Group, 2021). Continued MCE remains a priority, for example, during the development of recruitment, data collection, measurement protocols, and interpretation and dissemination of results.

It is worth emphasizing that, as previously mentioned, the framework is designed to be generalizable across different health inequities and requires further operationalization during the four-step application process. The framework’s value for designing and evaluating effective multilevel SDOH intervention is conditional on the critical appraisal and precise specification of its constructs and relationships in relation to a particular health inequity and context. To illustrate, we discuss an example for applying, operationalizing, and using the framework in the subsequent section and point the interested reader to literature discussing the framework in more depth than space allows here (Guilamo-Ramos et al., 2023; Thimm-Kaiser et al., 2023).

## An Exemplar Application of the Framework: Evaluating the Multilevel NCFP Intervention

In this section, we illustrate the utility of the CLAFH framework for guiding decisions across the development and evaluation of multilevel interventions using an exemplar application to the multilevel NCFP intervention (ClinicalTrials.gov, 2021), which is currently being evaluated as part of the NIH Rapid Acceleration of Diagnostics in Underserved Communities (RADx-UP) initiative focused on a community-engaged response to the inequitable impact of COVID-19 (Webb Hooper, 2022). NCFP is a nurse-driven, community-based intervention that leverages individual, family, institutional, and system factors to shape COVID-19 mitigation outcomes at the individual and household levels, including COVID-19 testing, vaccine uptake, and family mutual aid. NCFP is based on the principles of the nurse-led model of care (see Fig. S1 in the online supplement) and takes an approach focused on mitigating harmful SDOH. Photos showing NCFP implementation in the field are available online as a supplement to this article. NCFP illustrates how the heuristic framework can be applied in four steps to guide prevention research. We illustrate the application for each step below, with particular attention to step 4, evaluation.

### Step 1: Identification of a Specific Health Inequity and Context

NCFP was designed to address significantly elevated levels of COVID-19 morbidity and mortality in the South Bronx, New York City (NYC)—the health inequity and community context of interest for the current exemplar application of the framework. Since the first case of COVID-19 in NYC was documented in February 2020, the cumulative rate of COVID-19 deaths among Bronx residents has been nearly twice the national average (New York City Health, n.d.; Johns Hopkins University, n.d.-a), and the number of residents who have passed away from COVID-19 is higher in the Bronx than in 99% of the other counties in the USA (Johns Hopkins University, n.d.-b). The South Bronx, where the COVID-19 death rate surpasses that of Bronx County at large, lies in the poorest congressional district in the continental USA (United States Census Bureau, 2021) and is designated as a medically underserved area with a shortage of health professionals; 97% of all residents are Latino or Black, and 30% are foreign born (Hinterland et al., 2018). Similar communities persistently experience COVID-19 and other health inequities and represent contexts that warrant prioritization in efforts to develop and scale up multilevel interventions to address and mitigate the health impacts of structural racism and other harmful SDOH. The NCFP project leveraged the longstanding community partnerships of the investigative team and investigator positionality (i.e., MPI Guilamo-Ramos was born and raised in the Bronx and has conducted community-based research in the South Bronx for over 20 years), including with local health and social service providers, community leaders, tenant associations, and families, to inform all aspects of the study (Keene & Guilamo-Ramos, 2021).

### Step 2: Operationalization of the Framework Constructs, Mechanisms, and Leverage Points

**Fig. 2:**
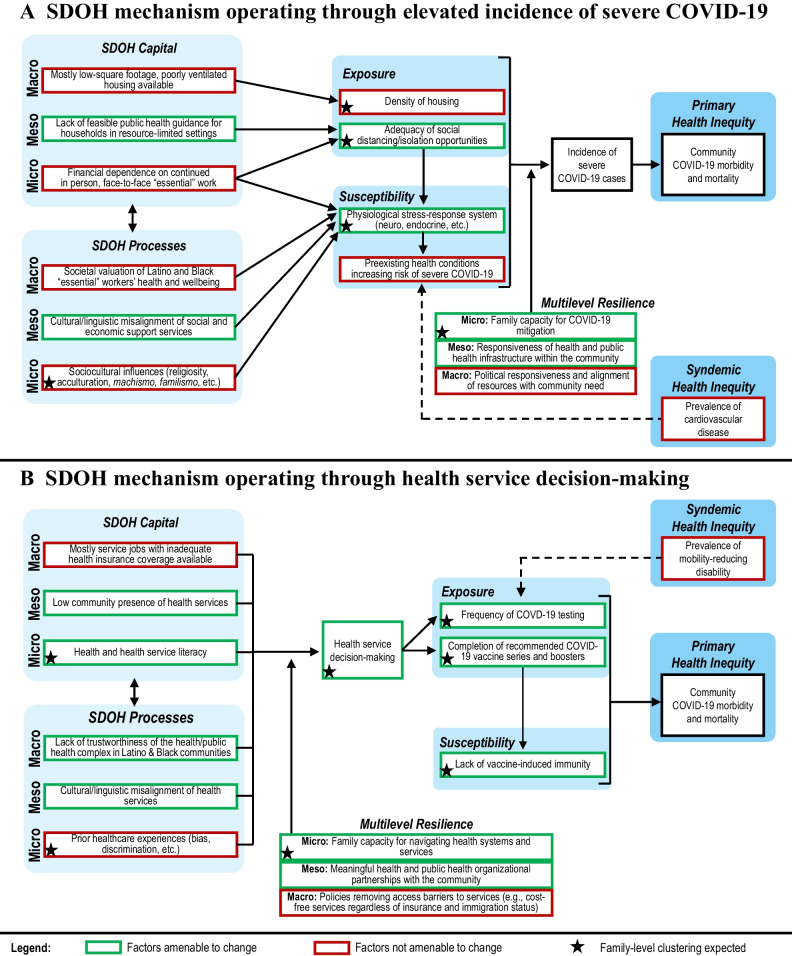
Operationalization of the CLAFH framework in relation to COVID-19 inequities in the South Bronx, NYC. *Note*. Social determinants of health influences that directly and indirectly shape health inequities through mechanisms that operate at multiple levels (i.e., micro, meso, and macro) and across the life course. Solid arrows reflect direct, mediated, and moderated relational forms, while dotted arrows represent the feedback effects of health inequities reinforcing harmful SDOH influences

Operationalization of the framework involves drawing on theory, empirical data, and published literature to define the constructs and relationships depicted in Fig. 1 in relation to COVID-19 inequities in the South Bronx (the specific inequity and context defined in step 1). The investigative team used the CLAFH framework as a roadmap to define two primary mechanisms through which SDOH capital and process factors shape COVID-19 morbidity and mortality inequities in the South Bronx (graphically depicted in Fig. 2A, B). Figure 2A describes multilevel SDOH factors shaping elevated rates of within-household COVID-19 transmission, particularly to household members at increased risk of developing severe COVID-19, as a mechanism driving COVID-19 inequities in the South Bronx. Figure 2B describes the mechanisms by which multilevel SDOH factors were hypothesized to shape the uptake of COVID-19 testing and vaccinations, in turn shaping community-level COVID-19 outcomes.

Notably, the hypothesized mechanisms of SDOH impact depicted in Fig. 2 distinguish between influences that were determined to be amenable to change within the context of the resources available to the NCFP project, therefore representing potential intervention levers (green) and those that were determined not to be amenable to change within the parameters of the NCFP project but the impact of which may be partially offset by the NCFP intervention (red). In this example, the operationalized CLAFH framework has utility for informing the design of the NCFP intervention by aligning NCFP components and content with the identified levers for intervention. Notably, operationalized framework pathways and constructs to be targeted by NCFP for mitigation of SDOH impact represent suitable primary and secondary outcomes for the subsequent NCFP evaluation study, given that the immediately measurable changes would be expected to occur in these variables. In addition, the operationalized framework can inform other aspects of the study measurement and analytic protocols, including selecting covariates for model specification and theory-based explanatory mediation analyses of intervention effects based on the hypothesized relationships depicted in Fig. 2.

### Step 3: Design of the Multilevel Intervention to Target the Identified Leverage Points

In step 3, the investigative team draws on the leverage points identified in step 2 to design a contextually tailored, multilevel SDOH mitigation intervention. Space constraints do not allow us to discuss each NCFP component in detail, but in short, the NCFP intervention was delivered via regular at-home support visits by nurse-community health worker (CHW) intervention teams, and its core active multilevel components include (1) at the individual level, behavioral intervention to shape COVID-19-related decisions (e.g., on testing and vaccine uptake) that draws on strong theories of health behavior; (2) at the family level, collaborative development of a tailored family infection control plan; (3) at the institutional level, transfer of the locus of healthcare to the household and community; and (4) at the systems level, formalized application of trustworthiness principles throughout all aspects of the NCFP intervention to enhance healthcare trustworthiness. The investigative team engaged its longstanding partnerships with key community gatekeepers (a resilience factor identified in Fig. 2), such as local tenant associations, during NCFP development. Table 2 presents select NCFP components, each corresponding to a leverage point identified during framework operationalization. A more detailed description of NCFP is available in the study protocol (ClinicalTrials.gov, 2021).

**Table 2:** NCFP intervention components addressing select amenable levers identified in Fig. 2

**NCFP intervention component**	**Leverage point identified in Fig.** 2
***Individual level***	
Regular at-home support visits by nurse-community health worker (CHW) intervention teams familiar with community-specific psychosocial challenges	Psychological stress response system
Culturally, linguistically, and contextually tailored NCFP workbook and community resource sheets	Health and health service literacy
Theory-based behavioral intervention to shape COVID-19-related decisions (e.g., on testing and vaccine uptake)	Health service decision-making
At-home offer of monthly routine and indicated COVID-19 testing (i.e., after exposure or symptoms)	Frequency of COVID-19 testing
In-person navigation to vaccine services	Completion of recommended COVID-19 vaccine series and boosters
Daily nurse-led symptom screening for triage and appropriate health service navigation of active COVID-19 cases	Lack of vaccine-induced immunity
***Family level***	
Collaborative development of a tailored family infection control plan	Adequacy of social distancing/isolation opportunities
Nurses deliver technical, knowledge, and skill training (e.g., training on household infection control skills and safe and correct use of PPE)	Family capacity for COVID-19 mitigation
Family mutual aid needs assessment and navigation to community-based support services	Family capacity for navigating health and social services and systems
***Institutional level***	
Transfer of the locus of healthcare to the household and community	Low community presence of health services
Bilingual/bicultural nurse-community health worker intervention teams; bilingual nurse hotline	Cultural/linguistic misalignment of health and social services
Screening for comorbidities and behavioral health needs; navigation to services where appropriate	Responsiveness of health and public health infrastructure within the community
Individualized household COVID-19 risk assessment and tailored guidance	Lack of feasible public health guidance for households in resource limited settings
Buy-in from CLAFH’s longstanding community partners (e.g., tenant associations) and collaboration in designing NCFP, including selection of the home-based nurse-community health worker-family partnership delivery model	Meaningful health and public health organizational partnerships with the community
***System level***	
Formalized application of four trustworthiness principles (transparency, respect, reliability, and benefit) throughout all aspects of the NCFP intervention	Lack of trustworthiness of the health and public health complex

### Step 4: Evaluation of the Multilevel Intervention

Our applied exemplar includes the real-world NCFP evaluation study, which has been in the field since July 2021 (ClinicalTrials.gov identifier: NCT04832919). We discuss this exemplar evaluation of a multilevel intervention with attention to the core characteristics of the overall study design, the selected analysis approach, and the approach to sample size calculation, highlighting decision points that were informed by the application of our heuristic framework. Finally, we discuss the strengths and limitations of the exemplar evaluation study.

#### Study Design

We employ a two-arm parallel explanatory group randomized trial (GRT) to evaluate NCFP outcomes at the (a) individual and (b) household levels. Several considerations emerging from steps 2 and 3 informed the selection of the GRT as the most appropriate design for the NCFP evaluation study. Based on the operationalized theoretical framework for the study, we anticipate family-level clustering for several key variables (indicated with a star in Fig. 2), including the primary (COVID-19 testing uptake) and secondary (adoption of COVID-19 control measures, COVID-19 vaccine uptake, mutual aid capacity, etc.) evaluation endpoints, which were selected in alignment with identified intervention leverage points. Therefore, the ability to accommodate individual- and family-level outcome evaluations was a primary consideration in selecting the GRT as an appropriate study design. In addition, the GRT represents the gold standard in trials in which the intervention (a) *operates at the group level* (i.e., NCFP is delivered in the home to households randomized to the experimental condition), (b) *manipulates the social or physical environment* (NCFP affects the family context in households participating in the intervention), or (c) *cannot be delivered to individuals without contamination* (family members within households receiving NCFP interact before and after randomization; Murray, 2015)—all three of which are the case in the current exemplar evaluation.

Using probability area sampling, we recruited a community-representative sample of 150 households (2–11 individuals per household) from public housing in the South Bronx, New York City (Hall, 2008). Households (groups) were randomized in a 2:1 ratio, intervention to control. NCFP was delivered to households over 5 months. The GRT assessments include theory-derived surveys ascertaining outcomes, hypothesized mediators and moderators, and other covariates (informed by the proposed framework) at baseline, monthly during months 1–6, and 9 months post-baseline. Surveys are completed by all consented individual household members aged 10 years and older. In addition, we formally assess measures of community engagement and racial/ethnic discrimination, with participants in the NCFP condition hypothesized to report increased perceived engagement and collaboration with the research team and the healthcare complex. Additional details are available in the study protocol (ClinicalTrials.gov, 2021).

#### Analysis Approach

For primary analyses, we will use a longitudinal random-coefficient logistic (binary outcome) or linear (continuous outcome) multilevel regression model (Moyer et al., 2022). Possible alternative analysis approaches for longitudinal parallel GRTs would have included, for example, constrained longitudinal data analysis (Lu, 2010) and marginal models using generalized estimating equations (Turner et al., 2017). Notably, selecting the analysis approach has important implications for defining the estimand and interpreting the trial results and should be made carefully. Given the novel and infectious nature of COVID-19, we hypothesized household-level clustering of several study outcomes (e.g., COVID-19 testing uptake, vaccine uptake, adoption of control measures, and mutual aid capacity; see Fig. 2) and chose a random-coefficient model, which has been shown to maintain the nominal type I error for longitudinal GRT data and which will give a conditional assessment of the time-varying intervention effects at the household-level after taking into account individual-level effects, assuming that household-level variation is normally and independently distributed with constant variance (Moyer et al., 2022). The basic generalized equation is

$$g\left({Y}_{ijkl}\right)=\mu +{C}_{k}+T{t}_{l}+{TC}_{k}{t}_{l}+{G}_{jk}+T{G}_{jk}{t}_{l}+{M}_{ijk}+{\varepsilon }_{ijkl},$$

where $g\left({Y}_{ijkl}\right)$ is a link function with $g\left({Y}_{ijkl}\right)\text{ = ln}\left[\frac{P\left({Y}_{ijkl}=1\right)}{1-P\left({Y}_{ijkl}=1\right)}\right]$ for a binary outcome (e.g., COVID-19 testing uptake) and $g\left({Y}_{ijkl}\right)={Y}_{ijkl}$ for a continuous outcome (e.g., adoption of COVID-19 control measures). $P\left({Y}_{ijkl}=1\right)$ is the conditional probability that the binary outcome equals one at assessment period *l* (*l* = 1, …, *t*; *t* = 8 for this study) for individual *i* (*i* = 1, …, *m*; *m* = 300 for this study) who resides in household *j* (*j* = 1, …, *h*; *h* = 150 for this study) that is assigned to condition *k* (*k* = 1, …, *c*; *c* = 2 for this study). $\mu$ is the log-odds (or mean) of the binary (or continuous) outcome in the control condition at baseline, and $C$_*k*_ is the baseline difference in log-odds for a binary outcome (or mean for a continuous outcome) between the experimental and control conditions (${C}_{1}=0$ for the control condition). $T$ is the fixed time slope in the control condition, ${t}_{l}$ is the value of time (continuous) at assessment period *l*, and ${TC}_{k}$ is the difference between the slope in the control condition and the experimental condition, so that the slope in the intervention arm is estimated as $T+{TC}_{k}$. ${G}_{jk}$ is the random intercept for household *j* in condition *k*, $T{G}_{jk}$ is the random time slope for household *j* in condition *k*, and ${M}_{ijk}$ is the random intercept for individual *i* who resides in household *j* in condition *k*.

We assume that the random effects are independent and normally distributed (Hox, 2002; Robinson, 2003). The variance partitioning coefficient, the amount of variability in the outcome attributed to predictors at each level, will be calculated. This will indicate the extent to which household- and individual-level effects shape the outcome, allowing for an examination of the impacts of the intervention at multiple levels (Draper, 1995; Gelman, 2012; Hswen et al., 2020). Among all the parameter estimates of fixed and random effects, the estimate of ${TC}_{k}$ is of our study’s main interest—the intervention effect over time after accounting for all other fixed and random effects. In addition, we will conduct sensitivity analyses (e.g., intent to treat vs. receive intervention and components of intervention received vs. per protocol) and subgroup analyses (e.g., race/ethnicity, gender, age, and education) based on findings from the primary analysis. Additional details are available in the study’s statistical analysis plan (ClinicalTrials.gov, 2021).

#### Sample Size Calculation

Consensus has not been determined on the precise power calculations for multilevel models (Maas & Hox, 2005; Snijders, 2005; Subramanian et al., 2003). We used Power Analysis and Sample Size software (PASS, 2023) to calculate power. PASS has a variety of functions designed to calculate power in mixed models for cluster randomized trials. We used procedures that were appropriate for our study design based on household-level 2:1 randomization, individual-level outcomes using conservative estimates, 15% attrition, and allowing subgroup analyses. We used a mixed model based on a clustered 3-level hierarchical design, where the repeated measures on participants represent level 1, individual participants represent level 2, and households represent level 3. Because most of the outcomes in this study are binary, we used a binary outcome as an example (for illustration) in this sample size calculation based on a test of two proportions in a 3-level hierarchical design with level 3 randomization. A sample size of 300 participants obtained from 100 level 3 units (households) in the intervention and 50 in the control group, with an average of two level 2 units (individuals) per level 3 unit and an average of eight level 1 units (time points) per level 2 unit, achieves > 80% power to detect a difference between group proportions of ≥ 0.15 at a significance level of 0.05 with the proportion in the control group as 0.10. The intraclass correlation coefficient of level 1 units within a level 2 unit is assumed as 0.7, of level 1 units within a level 3 unit is assumed as 0.5, and of level 2 units within a level 3 unit (intraclass correlation coefficient) is assumed as 0.3. Given the novel and infectious nature of COVID-19, we used conservative estimates for correlation in the a priori sample size calculation. There was limited data on this population or the outcomes of interests. We used higher-than-usual correlations in this data to accommodate potentially higher correlations among households, given that part of the intervention is creating a household plan. That said, we anticipate that correlation will be lower than we estimated a priori. We plan to do a post hoc power calculation after completion of data collection.

#### Strengths and Limitations of the Proposed Approach

Several strengths characterize the proposed approach to developing and evaluating multilevel interventions. First, our approach to intervention development, evaluation design, and analysis is informed by a strong theoretical framework that can be generalized for application to future multilevel interventions for contextually tailored mitigation of structural racism and other harmful SDOH across a range of health inequities. Second, we propose a rigorous explanatory household-level GRT design, which allows us to (a) make causal inferences on intervention effects (it should be noted that causal inference may not be appropriate for nonrandomized/observational research using our framework, depending on the statistical estimand and assumptions), (b) evaluate intervention effects at multiple levels (i.e., individual and household levels), (c) explain the mechanisms through which the intervention shapes outcomes in mediation analyses, and (d) maximize power and feasibility, given that we rely on a design with many groups (*n* = 150 households) and few individuals per group (~ 3 members per household). Third, we rely on a theory-based measurement protocol that permits covariate adjustment and mediation/moderation analyses and that formally assesses racial/ethnic discrimination and community engagement. Fourth, our analysis strategy accounts for variance and intraclass correlations due to group randomization and time effects. Fifth, the presented exemplar multilevel intervention and evaluation study is not hypothetical in nature, as the evaluation of NCFP is currently being implemented with funding from the NIH. Finally, the longstanding partnership between the investigative team and the South Bronx community has informed all aspects of the presented example.

The proposed approach also has some limitations. Given the substantial sample size demands of a factorial randomization design, our GRT relies on randomizing the multilevel components of the NCFP intervention as one package. While this approach makes the disaggregation of independent and synergistic effects attributable to specific intervention components analytically more complex, the proposed explanatory trial design accommodates mediation and moderation analyses, thereby balancing analytic rigor with practical considerations. Furthermore, our proposed analyses largely rely on participant self-reports collected in self-administered surveys, which may be subject to measurement, social desirability, and recall bias. However, the investigative team has extensive experience developing psychometrically sound measurement protocols for the study population. We assess a social desirability index to control for social desirability response tendencies, and the monthly survey assessments reduce the risk of recall problems. In addition, both social desirability and recall bias should occur equally in the experimental and control conditions. Last, we anticipate that the time points at which participants were enrolled in the study will influence their COVID-19 prevention behaviors, given that participants were enrolled over a multi-month period spanning several local COVID-19 waves and pandemic stages associated with changing public health guidance. However, we will analytically control for temporal effects, which should be equal across arms.

## Conclusions

The CLAFH framework is designed to serve as a generalizable template to aid in conceptualizing and operationalizing context-specific and multilevel SDOH mechanisms that shape a given health inequity, designing a multilevel intervention package tailored to target identified mitigation levers, and guiding the selection of an explanatory evaluation design, appropriate statistical methods, and approach to power calculations. The framework is a tool for researchers who seek to adopt a mechanism-focused approach to developing and evaluating multilevel health interventions that goes beyond establishing *whether* an intervention works and instead focuses on *how* an intervention works (Munson et al., 2022). Such approaches align with calls by the National Institute on Minority Health and Health Disparities (n.d.) and across other NIH institutes and offices (Blachman-Demner & Tyrus, 2022; NIH, 2023) for greater attention to the mechanisms through which structural racism and other SDOH shape health inequities. Our heuristic framework provides a conceptual roadmap for strengthening the scientific rigor of multilevel SDOH interventions. It constitutes an innovative addition to the available tools for applied and community-engaged research to advance health equity in communities affected by structural racism and other harmful SDOH.

### Supplementary Information

Below is the link to the electronic supplementary material.

Supplementary file1 (PDF 497 KB)
