# Supplementary material for: Application of a Heuristic Framework for Multilevel Interventions to Eliminate the Impact of Unjust Social Processes and Other Harmful Social Determinants of Health
Source: Prev Sci. 2024 Apr 12;25(Suppl 3):446–58. doi: 10.1007/s11121-024-01658-x (PMC11239765; doi:10.1007/s11121-024-01658-x)
Supplement: Supplementary file 1 — Supplementary file1 (PDF 497 KB) [file 11121_2024_1658_MOESM1_ESM.pdf]

Supplementary Materials for

Application of a Heuristic Framework for Multilevel Interventions to Eliminate the Impact of Unjust Social Processes and Other Harmful Social Determinants of Health

This PDF file includes:

Figure S1
Photos S1 – S2

Figure S1. The Center for Latino Adolescent and Family Health (CLAFH) Nurse-Led Model of Care

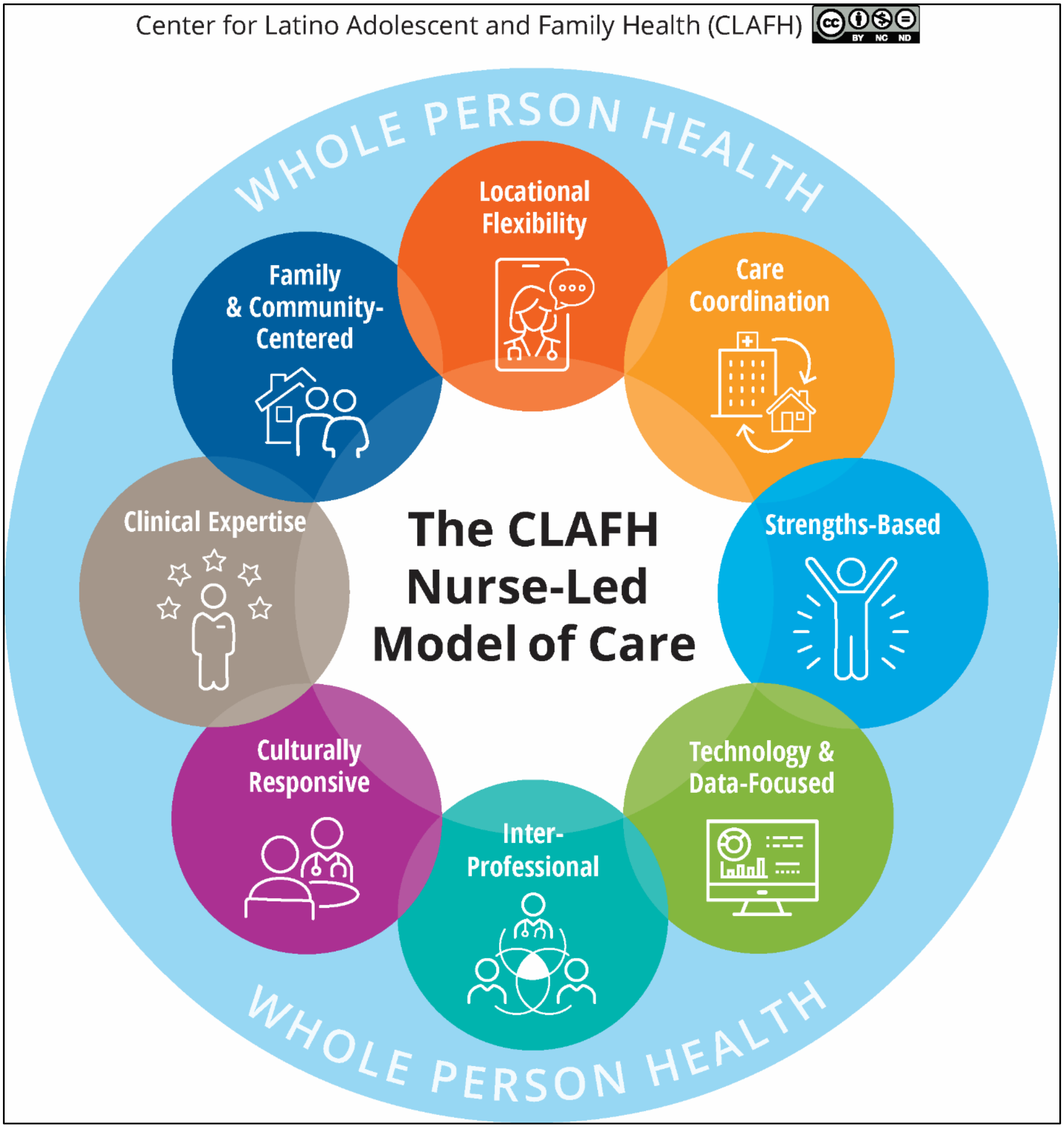

Photo S1.

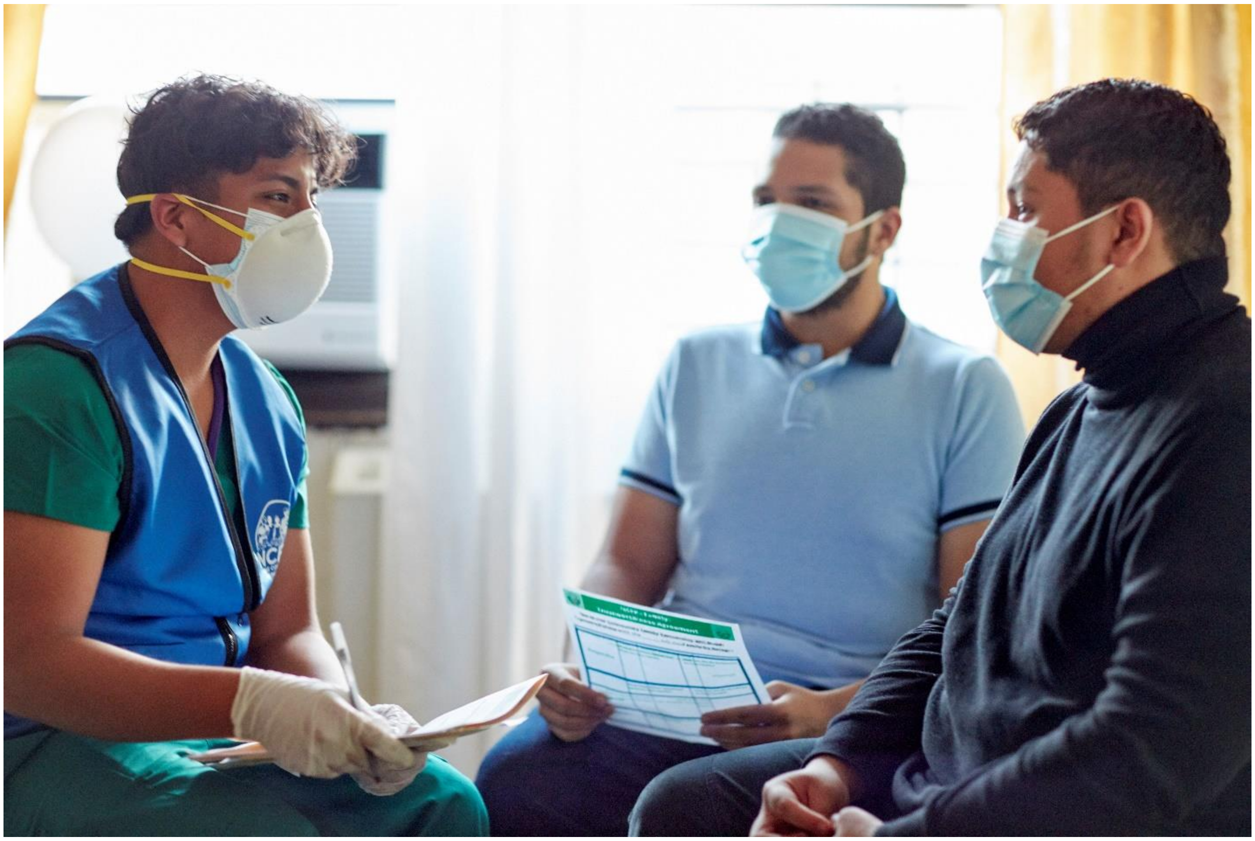

Photo Credit: Matthew Septimus

An NCFP community health worker (left) and family members collaboratively develop the NCFP Trustworthiness Agreement, an intervention element designed to foster meaningful community engagement.

Photo S2.

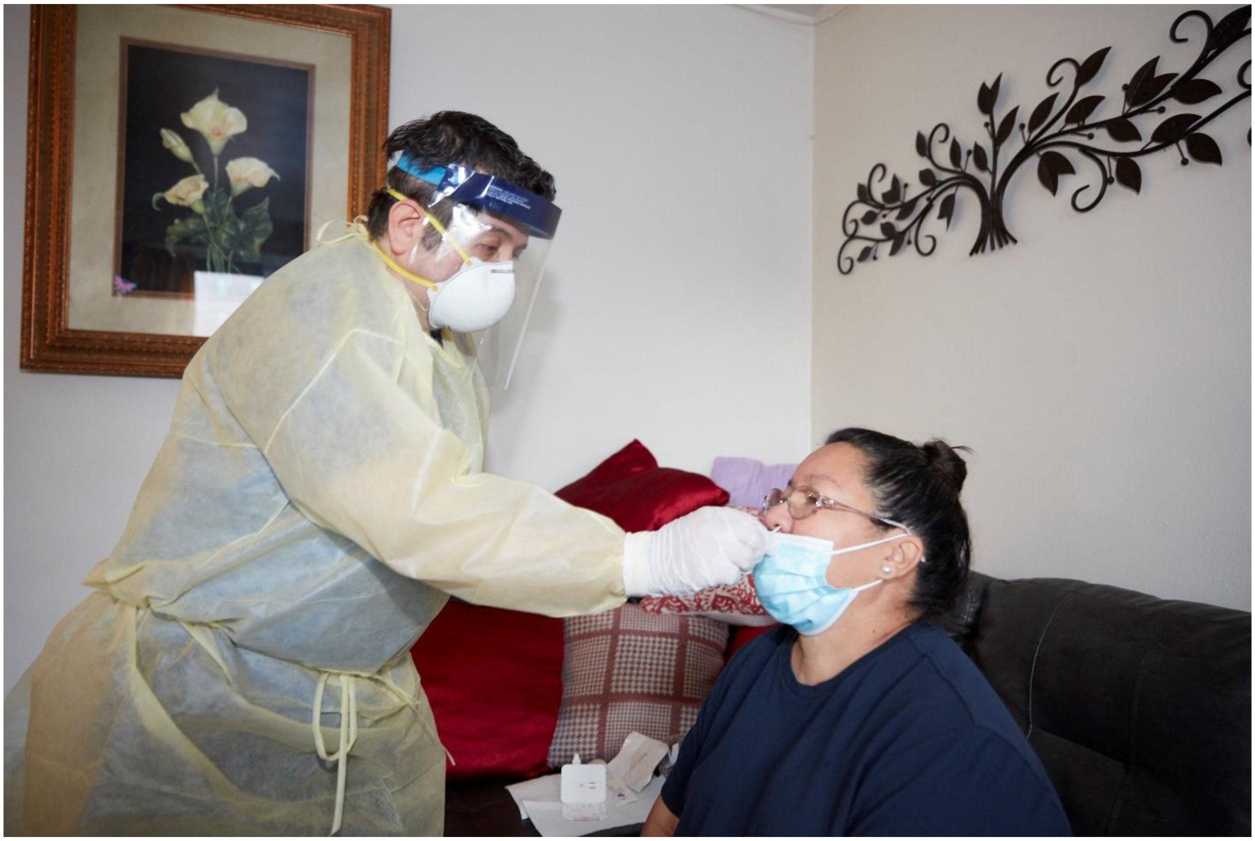

Photo Credit: Matthew Septimus

NCFP nurse collects a lower nasal swab for at-home rapid antigen COVID-19 testing (indicated testing, offered if a participant experiences symptoms consistent with COVID-19 or has had an exposure).
